# Multiple endocrine adverse reactions and pituitary axis dysfunction induced by PD-1 immunotherapy in an esophageal cancer patient: Case report

**DOI:** 10.1097/MD.0000000000048449

**Published:** 2026-05-22

**Authors:** Pei Sheng, Yuelin Guo, Yuqing Wu, Jing Xie, Xufang Wang, Mianhua Wu, Xiaofei An

**Affiliations:** a Department of Endocrinology, Affiliated Hospital of Nanjing University of Chinese Medicine, Jiangsu Province Hospital of Chinese Medicine, Nanjing, China; b Department of Oncology, Affiliated Hospital of Nanjing University of Chinese Medicine, Jiangsu Province Hospital of Chinese Medicine, Nanjing, China; c The First School of Clinical Medicine, Nanjing University of Chinese Medicine, Nanjing, China; d Department of Nephrology, Affiliated Hospital of Nanjing University of Chinese Medicine, Jiangsu Province Hospital of Chinese Medicine, Nanjing, China; e Jiangsu Collaborative Innovation Center of Traditional Chinese Medicine Prevention and Treatment of Tumor, Nanjing University of Chinese Medicine, Nanjing, China.

**Keywords:** autoimmune diabetes, hyponatremia, hypothyroidism, immune-related adverse events, multiple endocrine dysfunction, programmed death-1 inhibitor, secondary adrenal insufficiency

## Abstract

**Rationale:**

Immune checkpoint inhibitors, particularly programmed cell death protein 1 (PD-1) blockers, have transformed cancer therapy but can induce immune-related endocrine toxicities. Severe involvement of multiple endocrine axes, including dual pituitary dysfunction, is rare and challenging to diagnose due to nonspecific clinical features. Highlighting such cases can improve early recognition and management.

**Patient concerns:**

A 56-year-old man with esophageal cancer developed fatigue, polyuria, polydipsia, dizziness, and severe hyponatremia after treatment with tislelizumab, a PD-1 inhibitor.

**Diagnoses:**

Laboratory evaluation revealed new-onset autoimmune diabetes, hypothyroidism, and secondary adrenal insufficiency. The constellation of biochemical abnormalities and clinical features, in the absence of alternative etiologies, indicated immune-related hypophysitis and pancreatic islet injury. Two additional cases of severe, recurrent hyponatremia after PD-1 therapy were also reviewed.

**Interventions:**

The patient received targeted hormone replacement therapy, including glucocorticoids and levothyroxine, along with insulin for glycemic control.

**Outcomes:**

Symptoms improved significantly, and biochemical parameters normalized following treatment, demonstrating effective management of multi-glandular endocrine failure induced by PD-1 blockade.

**Lessons:**

PD-1 inhibitors can cause severe, concurrent dysfunction of multiple endocrine organs. Clinicians should maintain high vigilance, implement proactive screening for pituitary and metabolic abnormalities during and after immune checkpoint inhibitor therapy, and adopt a multidisciplinary approach to ensure timely diagnosis and treatment.

## 1. Introduction

Immune checkpoint inhibitors (ICIs) are monoclonal antibodies that block key regulatory signals suppressing immune responses. They function within 2 critical signaling pathways associated with T-cell activation and exhaustion by targeting cytotoxic T-lymphocyte antigen-4 (CTLA-4) or inhibiting the binding of programmed death-1 to its ligand (PD-1/PD-L1).^[1]^ ICIs have become standard therapy for multiple malignancies. While they eliminate tumors by modulating immune responses, excessively activated immune cells may induce autoimmune damage, termed immune-related adverse events (irAEs). With the widespread clinical application of ICIs, the incidence of irAEs continues to rise.

Endocrine irAEs represent the most prevalent category, occurring in approximately 40% of cases and affecting multiple organs. Commonly involved endocrine glands include the thyroid and pituitary, followed by the adrenals, pancreas, and parathyroids. Typical manifestations encompass thyroid dysfunction (hypothyroidism, hyperthyroidism, and thyroiditis), hypophysitis, hypopituitarism, primary adrenal insufficiency, type 1 diabetes mellitus, and autoimmune diabetes.^[1,2]^ Among patients receiving anti-PD-(L)1 therapy, hypothyroidism occurs in approximately 8%, hypophysitis in about 1%,^[3]^ and autoimmune diabetes in 0.2% to 1.4%.^[4–8]^ However, no previously reported cases describe the concurrent presentation of ICI-induced hypophysitis, secondary adrenal insufficiency, hypothyroidism, and autoimmune diabetes in a single patient.

This report details a middle-aged male with a history of esophageal squamous cell carcinoma who developed fatigue and anorexia following treatment with the PD-1 inhibitor tislelizumab. Based on his clinical course during and after PD-1 therapy, he was diagnosed with immune-related hypophysitis (IRH), anterior hypopituitarism, secondary adrenal insufficiency, hypothyroidism, and autoimmune diabetes. Additionally, we describe 2 other patients who developed hyponatremia as a manifestation of irAEs after PD-1 inhibitor therapy. Fatigue and anorexia are among the most common nonspecific adverse effects during anticancer treatment and are frequently overlooked, seldom linked to ICI-related toxicity. This case series aims to enhance clinical recognition of endocrine irAEs induced by PD-1/PD-L1 inhibitors and to challenge the habitual attribution of symptoms like fatigue and anorexia to conventional anticancer therapies alone.

## 2. Case report

A 56-year-old man presented to our hospital with the chief complaint of “generalized fatigue.” Upon admission, his associated symptoms included significant dry eyes, dry mouth, mild blurred vision, shoulder pain, lower limb weakness, foot numbness, loss of appetite, and polyuria. Two years prior, he was diagnosed with moderately to highly differentiated esophageal squamous cell carcinoma. His oncological treatment consisted of 3 cycles of neoadjuvant chemotherapy with the TC + PD-1 regimen (Paclitaxel for Injection (Albumin Bound) 400mg d1 + Carboplatin Injection 500mg d1 + Tislelizumab Injection 200mg d1), followed by radical esophagectomy. Postoperative pathology confirmed small residual tumor foci in the superficial muscle layer with no lymph node metastasis. He subsequently received 4 adjuvant cycles of the same TC + PD-1 regimen, followed by 12 cycles of Tislelizumab maintenance therapy from April 2023 to March 2024. During his immunotherapy, he developed elevated blood glucose and secondary hypothyroidism, which were not initially addressed. Six months prior to the current admission, he was diagnosed with “type 2 diabetes mellitus” and “hyperglycemic hyperosmolar state” (random glucose: 34.69 mmol/L) at a local hospital and was started on a insulin regimen. He had a 20-year history of smoking and alcohol consumption but had quit both.

## 3. Diagnostic assessment

**Figure 1.**
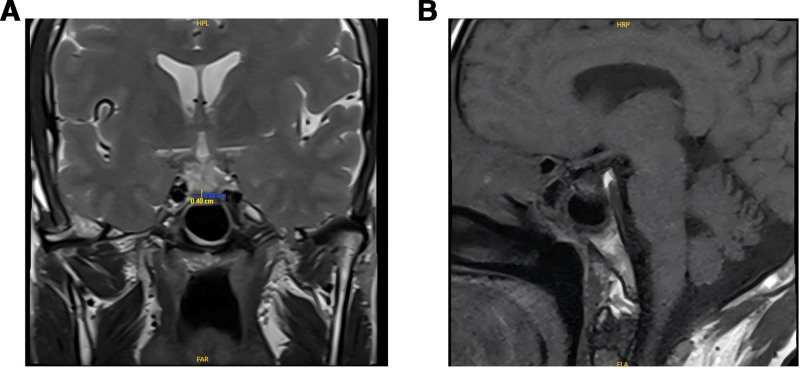
Pituitary MRI: (A) coronal; (B) sagittal. MRI = magnetic resonance imaging.

A systematic diagnostic workup was conducted to elucidate the etiology of the patient’s complex clinical presentation. The core findings were as follows. Dynamic monitoring of the pituitary-adrenal axis (Table 1) revealed a complete obliteration of the cortisol circadian rhythm, with critically low levels at all measured timepoints (<0.4 µg/dL at 8:00 am, 0.4 µg/dL at 16:00, and <0.4 µg/dL at 12:00 pm, reference: 6.7–22.6 µg/dL for 8:00 am). This was accompanied by profoundly suppressed ACTH levels (1.92 pg/mL at 8:00 am, <1.00 pg/mL at 16:00, and 1.07 pg/mL at 12:00 pm, reference: 7.20–63.30 pg/mL for 8:00 am), definitively confirming secondary adrenal insufficiency as the primary cause of hyponatremia. Assessment of the pituitary-thyroid (TSH) axis showed a pattern characteristic of central hypothyroidism, with a low free T4 (FT4) level of 0.55 ng/dL and a non-elevated TSH. Regarding pancreatic function (Table 2), the patient exhibited severe dysglycemia with a fasting glucose of 1.52 mmol/L and a 2-hour postprandial glucose of 15.38 mmol/L. Critically, both fasting and postprandial C-peptide levels were undetectable (<0.01 ng/mL), indicating a complete failure of endogenous insulin secretion. The fasting insulin level was low at 5.2 µIU/mL with an inadequate postprandial response (44.15 µIU/mL), and the absence of insulin autoantibodies supported the diagnosis of immune checkpoint inhibitor-induced autoimmune diabetes. Urine osmolality was 157 mOsm/kg, with a 24-hour urine volume of 3.1 L. 24-hour urine biochemistry showed a urine sodium level of 56.4 mmol/24 hour (more parameter details in Table 3). Further evaluation revealed concurrent secondary hypogonadism and insufficient growth hormone secretion (data not shown). Concerning electrolytes and urinalysis, the patient presented with severe hyponatremia (121.7 mmol/L) and hypochloremia (84.4 mmol/L), coupled with an inappropriately low urine osmolality (157 mOsm/kg) and a 24-hour urine sodium of 56.4 mmol/24 hour. This combination of findings was consistent with adrenal insufficiency and effectively ruled out the syndrome of inappropriate antidiuresis (SIADH). Imaging studies showed a slightly flattened pituitary gland on MRI, consistent with the later stages of IRH, while adrenal CT revealed no structural abnormalities (Fig. 1). Synthesizing this evidence, the final diagnosis was established as **PD-1 inhibitor-induced panhypopituitarism**, leading to secondary adrenal insufficiency and central hypothyroidism, co-occurring with **immune-related autoimmune diabetes**.

**Table 1:** Islet function test results.

Laboratory tests	Fasting	Normal range	2-h postprandial	Normal range
Insulin (µIU/mL)	5.2	1.90–23.00	44.15	–
C-peptide (ng/mL)	<0.01↓	0.78–5.19	<0.01↓	<6.73
Glucose (mmol/L)	1.52↓	3.89–6.11	15.38↑	<7.80

**Table 2:** Dynamic monitoring of diurnal rhythm variations of ACTH and cortisol in the blood.

Laboratory tests	8:00am	Normal range	16:00am	Normal range	12:00pm	Normal range
Serum cortisol (µg/dL)	<0.4↓	6.7–22.6	0.4↓	<10.0	<0.4	–
Adrenocorticotropioc hormone (pg/mL)	1.92↓	7.20–63.3	<1	–	1.07	–

ACTH = adrenocorticotropic hormone.

**Table 3:** Relevant laboratory test data.

Laboratory tests	Value	Normal range	Flags
Red blood cell count (10^12^/L)	3.61	4.00–5.50	Low
Hemoglobin (g/L)	116	120–160	Low
Aspartate aminotransferase (U/L)	18	<37	Normal
Alanine aminotransferase (U/L)	19	<41	Normal
Total protein (g/L)	60.91	64.00–83.00	Low
Prealbumin (mg/L)	62	125–400	Low
Albumin (g/L)	37.8	40.00–55.00	Low
Urea (mmol/L)	1.7	2.5–7.1	Low
Glucose (mmol/L)	12.46	4.10–5.90	High
Chloride (mmol/L)	84.4	98.0–107.0	Low
Magnesium (mmol/L)	0.64	0.70–1.00	Low
Sodium (mmol/L)	121.7	136.0–145.0	Low
Carbon dioxide	17.8	22–30	Low
High-density lipoprotein cholesterol (mmol/L)	0.86	0.90–1.90	Low
Low-density lipoprotein cholesterol (mmol/L)	1.31	2.07–3.10	Low
Insulin autoantibody (COI)	1.3	<0.9	High
Islet cell antibody (COI)	0.1	<0.9	Normal
Glutamic acid decarboxylase antibody (IU/mL)	1	<10	Normal
Tyrosine phosphatase antibody (IU/mL)	<0.70	<10	Normal
Zinc transporter 8 antibody (AU/mL)	<1.00	<10	Normal
Glycated hemoglobin (%)	12	4.0–6.0	High
Thyroid-stimulating hormone (µIU/mL)	8.321	0.560–5.910	High
Triiodothyronine (ng/mL)	0.61	0.66–1.61	Low
Total thyroxine (ng/mL)	106.1	60.9–122.3	Normal
Free triiodothyronine (pg/mL)	2.4	2.5–3.9	Low
Free thyroxine (ng/dL)	1.15	0.59–1.25	Normal
Urine creatinine (µmol/L)	2692	3450–22900	Low
24-h urine urea (mmol/24 h)	118	200–600	Low
24-h urine creatinine (µmol/24 h)	8345	7000–18000	Normal
24-h urine potassium (mmol/24 h)	16.7	25–100	Low
24-h urine sodium (mmol/24 h)	56.4	130–260	Low
24-h urine calcium (mmol/24 h)	2.05	2.5–8.8	Low
24-h urine uric acid (µmol/24 h)	1339	1500–4500	Low
24-h urine phosphate (mmol/24 h)	4.9	12.9–42.0	Low
24-h urine magnesium (mmol/24 h)	3.1	3.0–4.5	Normal
24-h urine glucose (mmol/24 h)	29.95	<0.08	High
24-h urine volume (L)	3.1	1.0–2.0	High
24-h urine protein (mg/24 h)	68	<150	Normal
Ferritin (ng/mL)	516.2	23.90–336.2	High
Carbohydrate antigen 199 (U/mL)	90.35	<37.00	High
Free prostate-specific antigen (ng/mL)	1.01	<0.420	High
Sex hormone-binding globulin (nmol/L)	152.3	11.2–78.1	High
Dehydroepiandrosterone sulfate (µg/dL)	40.1	136.2–591.9	Low
Testosterone (ng/dL)	724.72	175.00–781.00	Normal
Estradiol (ng/L)	52	20–47	High
Luteinizing hormone (mIU/mL)	3.91	1.24–8.62	Normal
Follicle-stimulating hormone (mIU/mL)	3.02	1.27–19.26	Normal
Progesterone (ng/mL)	0.25	0.14–2.06	Normal
Serum prolactin (ng/mL)	13.2	2.64–13.13	High
Insulin-like growth factor-1 (µg/L)	23	60–350	Low
Aldosterone (pg/mL)	93.39	10–160	Normal
Angiotensin II (pg/mL)	83.96	25–129	Normal
Renin (pg/mL)	35.63	2.4–32.8	High
24-h urine free cortisol (µg/24 h)	<12	58–403	Low
Serum growth hormone (ng/mL)	1.82	0.06–5	Normal

COI = cut-off index.

### 3.1. Therapeutic intervention and management outcomes

Based on the diagnosis, a comprehensive multi-hormone replacement regimen was initiated. The specific regimen included glucocorticoid replacement with prednisone acetate tablets, starting at 0.5 mg twice daily and promptly optimized to 5 mg in the morning and 2.5 mg in the afternoon to better mimic the physiological cortisol rhythm; thyroid hormone replacement with levothyroxine sodium tablets 150 µg once daily; and glycemic control via a basal-bolus insulin regimen comprising subcutaneous insulin glargine 14 IU in the morning and insulin aspart 14 IU, 14 IU, and 10 IU before meals.

### 3.2. Follow-up and treatment efficacy outcomes

The patient’s response to therapy was closely monitored and demonstrated significant improvement. In the short term, within 2 days of initiating glucocorticoid replacement, the patient reported a subjective improvement in generalized fatigue. Medium-term outcomes at the 2-month follow-up were more notably positive: the patient’s fatigue and loss of appetite were significantly resolved; the life-threatening hyponatremia was completely corrected, with serum sodium normalizing to 138.0 mmol/L; and thyroid function parameters normalized under replacement therapy (Table 4). Importantly, no recurrence of hyponatremia or other major immune-related adverse events was observed during the follow-up period, indicating stable endocrine management.

**Table 4:** Laboratory test results of the patient after 2 months.

Laboratory tests	Value	Normal range	Flags
C reactive protein (mg/L)	12.34	<8.00 mg/L	High
Red blood cell count (10^12^/L)	3.74	4.00–5.50	Low
Neutrophil percentage (%)	36	50.0–70.0	Low
Lymphocyte percentage (%)	41.6	20.0–40.0	High
Monocyte percentage (%)	9.1	3.0–8.0	High
Eosinophil percentage (%)	12.9	0.0–5.0	High
Absolute neutrophil count (10^9^/L)	1.73	2.00–7.50	Low
Absolute eosinophil count (10^9^/L)	0.62	0.00–0.50	High
Red cell distribution width (fL)	50.7	39.0–46.0	High
Aspartate aminotransferase (U/L)	18	<37	Normal
Alanine aminotransferase (U/L)	60	<41	High
Total protein (g/L)	66.43	64.00–83.00	Normal
Prealbumin (mg/L)	107	125–400	Low
Albumin (g/L)	41.5	40.00–55.00	Normal
Creatine kinase (U/L)	38	50–310	Low
Urea (mmol/L)	4.01	2.5–7.1	Normal
Glucose (mmol/L)	3.27	4.10–5.90	Low
Chloride (mmol/L)	102	98.0–107.0	Normal
Magnesium (mmol/L)	0.83	0.70–1.00	Normal
Sodium (mmol/L)	138	136.0–145.0	Normal
Thyroid-stimulating hormone (µIU/mL)	0.051	0.560–5.910	Low
Triiodothyronine (ng/mL)	0.99	0.66–1.61	Normal
Total thyroxine (ng/mL)	102.4	60.9–122.3	Normal
Free triiodothyronine (pg/mL)	3.7	2.5–3.9	Normal
Free thyroxine (ng/dL)	1.18	0.59–1.25	Normal
Squamous cell carcinoma antigen (ng/mL)	4.5	<1.5	High

### 3.3. Outcomes of supplementary cases: highlighting the spectrum of hyponatremia

The outcomes of the 2 supplementary cases further illustrated the varied clinical spectrum of PD-1 inhibitor-associated hyponatremia. Patient 2 (treated with Tislelizumab) demonstrated the challenge of recurrent, refractory hyponatremia, which persisted despite intermittent sodium supplementation and low-dose steroid therapy. The management was complicated by the presence of hypothalamic metastases. This case underscores that concurrent central nervous system disease can confound the diagnosis and management of ICI-related hyponatremia. In contrast, Patient 3 (treated with Toripalimab) presented a striking example of severe but highly glucocorticoid-responsive hyponatremia. The patient’s serum sodium dropped to 115.6 mmol/L and was refractory to sodium supplementation alone (Table 5). However, it showed a dramatic and rapid correction to 141.4 mmol/L following high-dose intravenous hydrocortisone pulse therapy. This unequivocal response confirmed the diagnosis of ICI-induced adrenal insufficiency and highlights the critical importance of targeted hormone replacement over mere sodium supplementation.

**Table 5:** Dynamic monitoring of diurnal rhythm variations of ACTH and cortisol in the blood of patient 3.

Laboratory tests	8:00am	Normal range	16:00am	Normal range	12:00pm	Normal range
Serum cortisol (µg/dL)	<0.4↓	6.7–22.6	0.4↓	<10.0	<0.4	1–
Adrenocorticotropioc hormone (pg/mL)	<1↓	7.20–63.3	<1	–	<1	–

ACTH = adrenocorticotropic hormone.

## 4. Discussion

We report a rare case of multiple endocrine gland injuries with dual pituitary axis impairment induced by the PD-1 inhibitor tislelizumab, alongside 2 additional cases of severe hyponatremia related to PD-1 blockade. These grade 3 to 4 immune-related adverse events (irAEs) are uncommon in their concurrent presentation, as most reported endocrine irAEs manifest as isolated disorders such as adrenal insufficiency or hypothyroidism. The nonspecific nature of symptoms and the potential for onset during therapy or months after cessation underscore the need for heightened clinical vigilance.^[9]^

Per ASCO guidelines,^[9]^ baseline endocrine assessment prior to immune checkpoint inhibitor (ICPI) initiation is essential. Our index patient had no prior steroid exposure, renal disease, gastrointestinal disorders, or other secondary causes. The combination of multiple hormonal deficiencies, pituitary MRI changes, and the absence of alternative etiologies supports a diagnosis of PD-1–related immune-mediated hypophysitis. Hypophysitis occurs more frequently with CTLA-4 inhibitors (~3.2%) than with PD-1 (0.4%) or PD-L1 (<0.1%) agents,^[10]^ with combination therapy increasing risk. The timing of onset differs among ICPI types: median 30 days with combination therapy, 2 to 3 months with CTLA-4 inhibitors, and 3 to 5 months with PD-1/PD-L1 agents.^[11,12]^ PD-1/PD-L1–related hypophysitis shows male predominance in patients over 60 years,^[13]^ with higher CTLA-4 dosing correlating with increased incidence.^[14]^

The pathogenesis of IRH remains incompletely understood. CTLA-4 inhibitors may trigger type II hypersensitivity–mediated pituitary autoimmunity,^[15]^ whereas the mechanisms underlying PD-1–related hypophysitis are less clear. Diagnostic criteria include ICPI exposure; new-onset deficiency of ≥1 pituitary hormone (TSH or ACTH required) with MRI abnormalities, or ≥2 deficiencies accompanied by symptoms such as headache regardless of imaging.^[16]^ Pituitary MRI may reveal enlargement, stalk thickening, or heterogeneous enhancement in ~77% of cases, though 23% to 33% present with normal imaging.^[17,18]^ Some patients may progress from normal MRI to pituitary atrophy over time.^[19]^ PD-1/PD-L1 inhibitor-related hypophysitis typically manifests as isolated ACTH deficiency^[20–22]^ and often lacks pituitary enlargement,^[23–26]^ in contrast to CTLA-4-related disease, which commonly involves multiple hormone axes.^[27]^ Headache with pituitary enlargement favors CTLA-4-related etiology.^[23]^ Because imaging changes may lag behind clinical onset,^[10]^ normal MRI does not exclude IRH.^[26]^

Endocrine irAEs may emerge or persist long after ICPI discontinuation,^[28,29]^ likely reflecting prolonged immune activation.^[29,30]^ In our series, 1 patient developed refractory hyponatremia from autoimmune hypophysitis 14 months post-therapy,which resolved only after glucocorticoid administration, highlighting the need for ongoing vigilance. Diagnosis is further complicated by the absence of specific biomarkers and overlapping clinical presentations.^[31–33]^ Potential biomarkers include anti-pituitary antibodies, detectable in isolated ACTH deficiency or hypophysitis without enlargement^[34,35]^; HLA variants (HLA-DR15, HLA-B52, HLA-Cw12) enriched in patients with hypothalamic-pituitary irAEs^[36,37]^; and antihypothalamus antibodies (AHA), which may precede anti-pituitary antibodies, implicating hypothalamic autoimmunity.^[38]^ Radiotherapy may exacerbate hypophysitis, as suggested in Patient 2, though confounding factors limit definitive conclusions.

Before initiating ICPI therapy, clinicians should document endocrine and autoimmune history, assess baseline function, monitor endocrine parameters, and maintain a high index of suspicion for irAEs when new symptoms arise.^[39]^ Patient education regarding early symptom recognition is crucial.^[40]^ In our series, delayed recognition in Patient 1 – initially misdiagnosed as diabetes – underscores the importance of considering ICPI exposure in differential diagnoses, especially for nonspecific complaints. As immunotherapy use expands, awareness of atypical and delayed endocrine irAEs, multi-axis involvement, and the potential for persistent dysfunction is essential for timely diagnosis and optimal patient outcomes.

This report has several limitations. First, as a single-center case series with a small number of patients, generalizability is limited. The findings are descriptive and cannot determine the true incidence or prevalence of multi-glandular endocrine dysfunction in the broader population. Second, the diagnosis of IRH was based on clinical, biochemical, and imaging findings; pituitary biopsy was not performed due to its invasiveness and associated risks. Third, follow-up duration was relatively short, limiting insight into long-term recovery of pituitary or pancreatic islet function. Finally, we cannot entirely exclude potential contributions from prior chemotherapy or the underlying malignancy, although the temporal relationship strongly implicates the PD-1 inhibitor as the primary cause. Future multi-center studies with larger cohorts and longer follow-up are needed to better define risk factors, natural history, and optimal management strategies for these complex endocrine irAEs.

## Author contributions

**Conceptualization:** Pei Sheng, Xufang Wang, Xiaofei An.

**Data curation:** Pei Sheng, Yuelin Guo.

**Funding acquisition:** Xiaofei An.

**Investigation:** Mianhua Wu.

**Methodology:** Pei Sheng, Yuqing Wu, Jing Xie.

**Project administration:** Pei Sheng, Xiaofei An.

**Resources:** Yuqing Wu.

**Software:** Yuelin Guo, Jing Xie.

**Supervision:** Jing Xie.

**Writing – original draft:** Pei Sheng, Yuelin Guo, Xufang Wang.

**Writing – review & editing:** Mianhua Wu, Xiaofei An.
